# Interspecies Communication and Periodontal Disease

**DOI:** 10.1155/2013/765434

**Published:** 2013-12-10

**Authors:** Ajay Mahajan, Baljeet Singh, Divya Kashyap, Amit Kumar, Poonam Mahajan

**Affiliations:** ^1^Department of Periodontics, Himachal Pradesh Government Dental College and Hospital (H.P.G.D.C.), Shimla, Himachal Pradesh 171001, India; ^2^Himachal Pradesh Government Dental College and Hospital (H.P.G.D.C.), Shimla, Himachal Pradesh 171001, India

## Abstract

More than 500 bacterial strains may be found in dental plaque. In the beginning, the emphasis was laid on the isolation of bacteria in pure culture to define their properties. However, now, it has been well established that in nature the bacteria exist as a member of polymicrobial community or consortium of interacting species. Interactions among human oral bacteria are integral to the development and maturation of the plaque. These interactions occur at several levels including physical contact, metabolic exchange, small-signal molecule-mediated communication, and exchange of genetic material. This high level of interspecies interaction benefits the microorganism by providing a broader habitat range, effective metabolism, increasing the resistance to host defence, and enhancing their virulence. This generally has a detrimental effect on the host and is attributed to many chronic infections which poses a therapeutic challenge.

## 1. Introduction

Since the beginning of civilisation, mankind has been trying hard to understand and treat various forms of diseases which pose a danger on the survival of human race. Before the invention of the microscope, little was known about the etiology of the disease as a result of which the treatment approach as well as options were limited. Anton van Leeuwenhoek was the first scientist who observed microorganisms with a microscope in 1683 [1] and with this the era of microbiology began. One of the first samples he examined was his own dental plaque or biofilm. The major breakthrough came when a French chemist, Louis Pasteur, and a German physician, Koch, established the concept that bacteria caused the disease. W. D. Miller is credited for establishing the role of microorganisms in the etiology of the oral disease. Existence of plaque was for the first time described in 1897 by James Leon William and the term was coined by G. V. Black.

The development in the microscopic techniques allowed the researchers to do more accurate and sophisticated analysis. Initially, descriptive histological analysis was done, but now the emphasis is laid on identifying the changes at the cellular and molecular level. Researchers have done a lot and are still working hard to better understand the microorganisms and their etiopathological role in various forms of human diseases. Studies have now established the fact that microorganisms in plaque do not exist as independent units but as a highly complex interacting colony—the biofilm. Within the biofilm, microorganisms not only get a favorable environment for growth but are also protected from the antibacterial substances. The role of plaque in the aetiology of the periodontal disease has been established beyond doubt. The Interspecies communication within the biofilm plays a major role in the initiation and progression of the periodontal disease. Therefore, understanding the mechanical and chemical interaction between the microorganisms becomes indispensable to formulate a successful treatment regime for the periodontal disease.

## 2. Biofilms

Biofilms are defined as oriented aggregations of microorganisms attached to each other or to a surface and enclosed in extracellular polymeric substance produced by themselves [2, 3].

**Figure 1:**
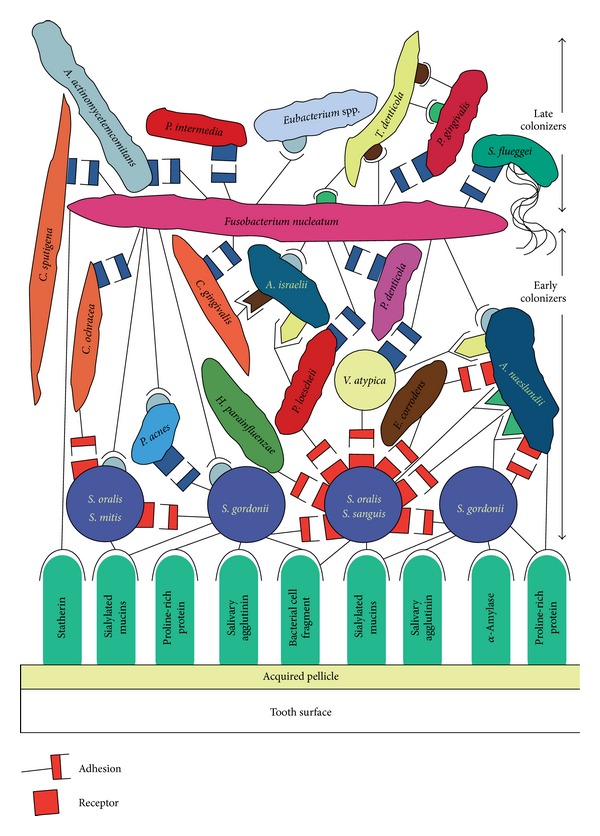
Showing the formation and maturation of dental plaque. (Source [4] ).

*Formation and Maturation of Oral Biofilm.* Biofilm formation is a very ordered and coordinated process, involving sequential formation of acquired pellicle, colonisation of primary colonisers and then secondary colonisers (Figure 1). In the absence of communication, these orderly changes would be random [4]. Researchers now have substantial data to prove that bacteria communicate to accomplish ordered multispecies communities.

The acquired pellicle, which is composed of a variety of host-derived molecules, coats the enamel surface within minutes after professional cleaning and is a source of receptors for primary colonisers of the plaque [4]—*Streptococci* spp., *Actinomyces* spp., *Capnocytophaga* spp., *Eikenella* spp., *Haemophilus* spp., and *Veillonella* spp. As soon as the pioneer bacteria attach on the pellicle, they begin to excrete extracellular polymeric substance, which helps the bacteria stay bound together and attach to the pellicle [5]. The pioneer bacteria provide specific binding sites for the subsequent bacterial colonisation. Bacteria coaggregate, forming typical corncob forms, bristle brush form, or other forms in mature oral biofilm. Coaggregation is defined as the specific cell-to-cell recognition that occurs between genetically distinct cell types. Each cell type bears on its surface one or more types of coaggregation mediators, which are called adhesions and receptors. Coaggregation effect changes in populations from low diversity in initial populations of supragingival plaque to high diversity in subgingival [3]. Subsequent attached bacterial species include *Fusobacterium nucleatum, Treponema* spp., *P. Gingivalis* spp., and *Aggregatibacter actinomycetemcomitans* [6]. As the biofilm matures, there is proportional shift in the microbial components. The relative amount of *Streptococci* and *Neisseria* decreases, while the amount of *Actinomyces, Corynebacterium, Fusobacterium, and Veillonella* increases.

*Fusobacterium nucleatum* is thought to play a central role in the maturation with almost all bacteria including early colonisers and later colonisers [7]. Thus, *Fusobacterium nucleatum* forms a coaggregation bridge between the bacteria which do not naturally coaggregate with each other.

## 3. Communication

Communication is a key element in successful organisations. Numerous studies have shown that a very sophisticated communication system exists in the oral biofilm. There are over 500 different bacteria species in the oral microflora. Some of these species are considered commensal and a positive feature of our healthy microflora, while other are considered pathogenic. It is unlikely that various species within oral biofilms function as independent, discrete constituents rather, these organisms function as a coordinated community that uses intra- and interspecies communication [4].

*Why Do They Need Communication?* Effective communication between workers is essential for the smooth running of the operation. In the same way, bacteria building oral biofilms adopt specialized roles and communicate with one another. Throughout the development of dental plaque, adherent bacteria sense their neighbors and make appropriate responses (Table 1).

## 4. Mechanical and Chemical Communication in Oral Biofilm

**Figure 2:**
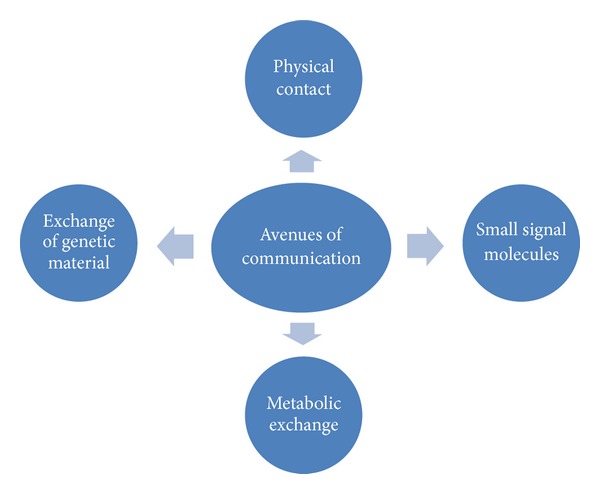
Showing various methods of communication among microorganisms in biofilm.

Various modalities of communication among microorganisms in oral biofilm are, as shown in Figure 2,

1. physical contact,
2. metabolic communication,
3. small signal communication,
4. genetic exchange.

*(1) Physical Communication. *It plays a major role in the formation and maturation of dental plaque. Acquired pellicle coats the surface and acts as a source of receptors for the primary colonisers of the plaque. The primary colonisers provide the specific binding site for the secondary colonisers.

*(2) Metabolic Communication*. The foundation of biofilm metabolic communication is coaggregation. Bacteria sense the changes in the local environment that are caused by neighboring cells and in this way receive information about the surrounding microbial population. Information about the sender is sent to the receiver and the result is altered gene expression and phenotype.

Examples of various metabolic communications as follow.

**Scheme 1:**
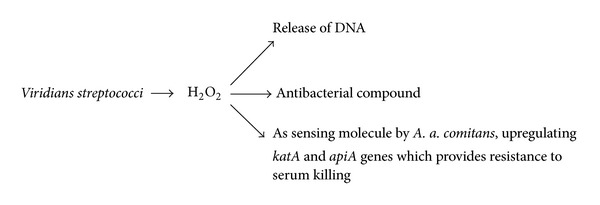


**Scheme 2:**
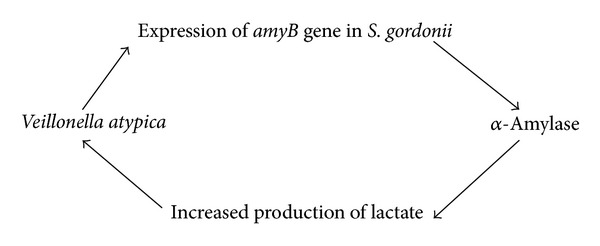


**Scheme 3:**
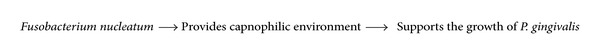


**Scheme 4:**
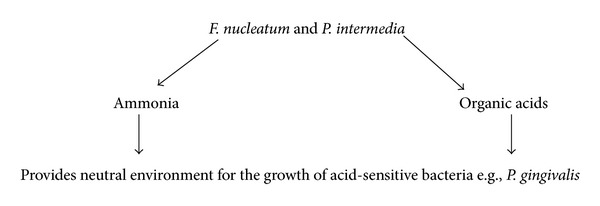


**Scheme 5:**
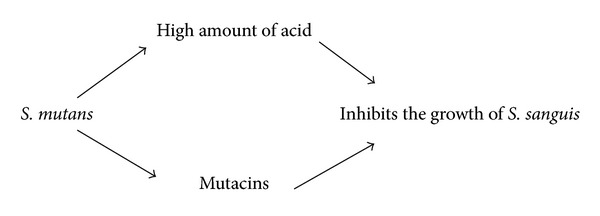


**Scheme 6:**
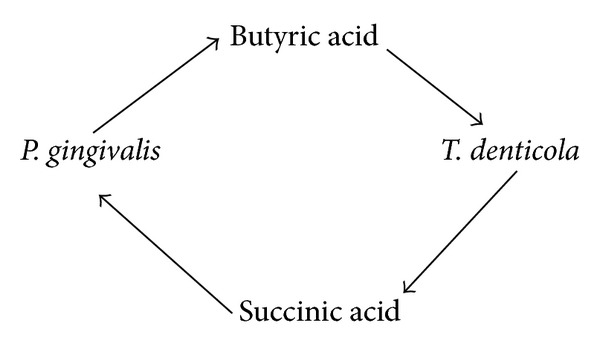


**Scheme 7:**
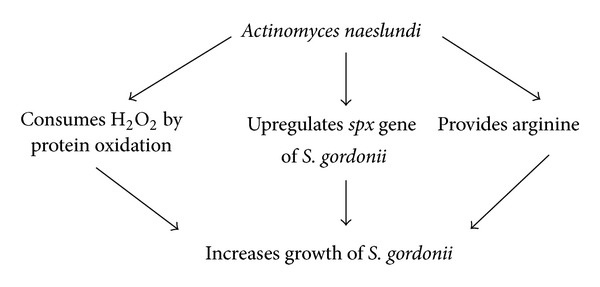


**Scheme 8:**
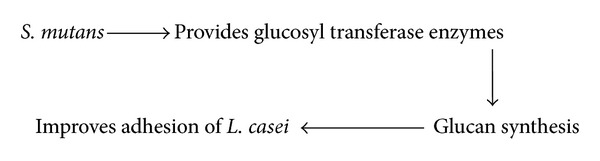


- *Streptococcal H*_*2*_ *O*_*2*_ is an important molecule in the competition and communication between oral bacteria [8, 9] (see Scheme 1).
- Interspecies communication between oral *streptococci and veillonella* appears to be driven by metabolic requirements [10] (see Scheme 2).
- Oxygen metabolism and exchange: it is difficult for obligate anaerobic species to live in an environment without the cooperation of aerobic species [5] (see Scheme 3).
- Some bacterial species can help other species survive by depleting acid and improving the local environment (see Scheme 4).
- The numbers of *S. mutans and S. sanguis* are negatively associated. it has been found that *S. mutans* inhibits the growth of *S. sangius* by producing large amount of organic acid and mutacin [11] (see Scheme 5).
- A cooperative synergistic growth relationship exists between *P. gingivalis* and *T. denticola* [12] (see Scheme 6).
- *Actinomyces naeslundii* is a pioneer bacteria in dental plaque which protects many bacterial species from hydrogen peroxide (see Scheme 7).
- *S. mutans *provides glucosyl transferase enzymes, *L. casei*, which improves its adhesion by glucan synthesis (see Scheme 8).

**Figure 3:**
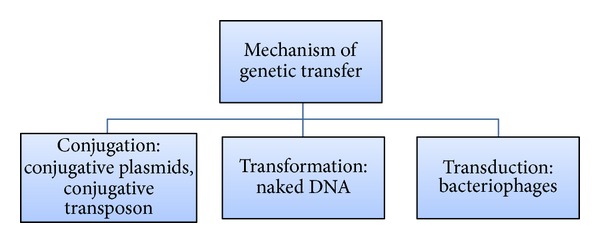
Showing different mechanisms of genetic transfer.

*(3) Horizontal Gene Tranfer. *It is recognised as a major contributor in the molecular evolution of many bacterial genomes [13]. The dense population structure in biofilms increases the opportunity of gene transfer between the species which can convert a previously avirulent pathogen to a highly virulent pathogen (see Figure 3). Evidence of horizontal gene transfer is provided by the presence of conjugative transposons and insertion sequence among various bacterial species, for example, *S. mutans* showing the presence of TnSmu1 transposon.

*Conjugation*. It is the polar transfer of genetic material through direct cell-to-cell contact, for example,

- tet(B) gene (providing tetracycline resistance) transfer between the strains of *A. comitans* and between *A. comitans and H. influenza*.
- pMER1 (plasmid-conferring mercury and streptomycin resistance) has been recently orally isolated.
- Tn916, encodding for antibiotic resistance, transfer among oral streptococci.

*Transformation.* It is defined as the uptake and maintenance of DNA, for example,

- erythromycin resistance gene transfer among *S. mutans* [14],
- intergeneric transfer between *T. denticola* and *s. gordonii*.

*Transduction*. It is genetic transfer through bacteriophages, leading to lysogenic conversion of many nonpathogenic bacteria, for example, Tn*916* and pkT210 transfer between strains of *A. comitans* by Aaø23 phage [15].

*(4) Signalling Chemical Agents and Quorum Sensing*. Quorum sensing (QS) is the self-induced secretion of one/more agents in response to changes in bacterial density and the surrounding environment which initiates gene expression to regulate cell or group behaviour. QS Systems control a wide range of responses including bacterial surface adhesion, extracellular matrix production, and competency [5].

Autoinducer-2* (AI-2)* is an important signal molecule in multispecies and plays a decisive promoting role in the formation and maturation of plaque. AI-2 is an umbrella designation that covers a collection of molecules formed from the spontaneous rearrangement of 4,5-dihydroxy-2,3-pentanedione (DPD), which is a product of LuxS. LuxS is present in the genome sequences of many oral bacteria, including, *S. mutans*, *S. gordonii*, *S. oralis*, and *P. gingivalis* [16]. AI-2 induces a widespread changes in gene expression. Evidences support that AI-2 acts both as intra- and interspecies signal. A study demonstrated that *P. gingivalis* can detect AI-2 signal from *Aggregatibacter actinomycetemcomitans*, thus providing the first evidence of interspecies communication using AI-2 [17]. An important function of AI-2 in mutualistic association was demonstrated in studies of dual-species biofilms formed with *A. oris and S. oralis* [18]. Commensal oral bacteria respond to low levels of AI-2, whereas periodontopathogenic bacteria respond to higher levels of AI-2. With the maturation of plaque, bacteria send and receive AI-2 at a much higher levels; there is a decrease in the growth of commensal bacteria [19].

Competence stimulating peptide *(CSPs)* regulates genetic competence, biofilm formation, and acid tolerance of bacteria. CSPs are short peptides, approximately 17–21 amino acids, produced by many Streptococci from the proteolytic digestion of the *comC gene* product [20]. CSPs can induce alarmones which are intracellular signal molecules and are produced due to harsh environmental factors and can convey sophisticated messages. CSPs have diverse effect on oral streptococci including promoting competence, biofilm formation, and DNA release [21]. CSP-sensing pathway in *S. mutans* is linked to the production of mutacins and bacteriocins with antimicrobial activity against a range of oral bacteria. CSP molecules are highly species specific. A CSP produced by one bacterium rarely interferes with the activity of CSP produced by a different bacterium. Eckert et al. have developed a highly novel antibacterial agent using the species-specific nature of CSP, called Specifically Targeted Antimicrobial peptides* (STAMPS)*, containing two functional domains, the first domain is a targeting peptide domain and the second is an antimicrobial peptide domain.

## 5. Interspecies Communication and Periodontal Disease

**Figure 4:**
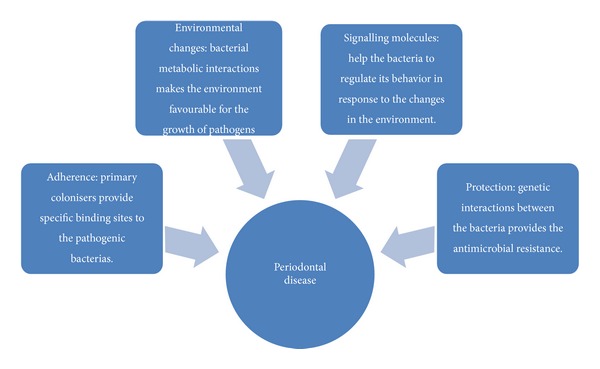
Showing the relation between the interspecies communication and the periodontal disease.

Periodontal diseases are mainly caused by dental plaque which is “specific but of highly variable structural entity resulting from sequential colonization and growth of microorganisms on the surfaces of teeth and restoration consisting of microorganisms of various strains and species is embedded in the extracellular matrix, composed of bacterial metabolic products and substances from serum, saliva, and blood” (WHO, 1978). Microorganisms in dental plaque are found in “complexes,” based on the frequency of which microorganisms are recovered together. Different microbial complexes have now been identified and are believed to be associated with various stages of disease initiation and progression. Members of the yellow complexes (*Streptococcus* spp.) and the purple complexes (*A. odontolyticus and V. parvula*) are the early colonisers of the dental plaque. Members of the green (*E. corrodens, A. a. comitans*,* and Capnocytophaga* spp.), orange (*Fusobacterium, Prevotella, and Campylobacter* spp.), and red (*P. gingivalis, B. forsythus, and T. denticola*) are the secondary colonisers of the dental plaque. Members of green and orange complexes have been recognised to be pathogenic causing certain periodontal and nonperiodontal infections. The red complex is associated with bleeding on probing. Bacteria of the orange complex may also be associated with red complex microorganisms, found in greater numbers in diseased sites and in more advanced periodontal disease. Thus, there exists interdependency among the bacteria present in the same or different complexes, for which an effective communication mechanism is essential. Communication among different bacterial species improves their chance of survival and thus has a major role in the etiopathology of the periodontal disease. Physical communication provides the site for adherence to the successive microorganisms; metabolic communications bring about the environmental changes favorable for the growth of pathogens; signaling molecules help bacteria to regulate their behaviour in response to changes in the environment; and genetic communication provides the microbial resistance against the antibiotics. Interspecies communication plays a major role in the initiation and progression of periodontal disease (see Figure 4).

## 6. Summary and Conclusion

Biofilm is a very systematic unit in which the microorganisms work in a well-coordinated manner. Interspecies communication plays a pivotal role in the formation, growth, and maturation of the biofilm. When assessing the ability of oral bacteria to cause disease, it is essential to consider the community in its entirety rather than solely on the observations of individual components. Understanding and exploring the interspecies communication will open the new era of periodontal therapy. By specifically promoting and inhibiting these interactions, we can control the microbial structure of the oral biofilm. Oral health could be achieved by promoting the interactions between the health-promoting bacteria, while the development and progression of the periodontal disease can be inhibited by blocking the interaction among the periodontal pathogens. However, much research is required before we can enter this fascinating therapeutic regime. Hopes are high with the emergence of latest microbiological and genetic innovations in the field. The paper elaborates various mechanical and chemical interspecies interactions and their role in the initiation and progression of periodontal disease.

## Figures and Tables

**Table 1:** Describing the various benefits of interspecies communications in biofilms.

Communication	
Increase chances of survival	Protection from environment
(i) Primary colonisers provide site for the adherence of the secondary colonisers. (ii) Coaggregation* bridge* allows anaerobes to come together and interact which normally do not coaggregate. (iii) Metabolic interactions which makes the biofilm environment favourable for the survival of bacterial species. (iv) Signaling molecules to regulate cell behavior in response to environmental changes. (v) Genetic exchange increases the pathogenicity of microbes.	(i) Extracellular polymorphic substance restricts the diffusion of antimicrobials from the surrounding environment. (ii) Horizontal gene transfer which provides antibiotic resistance.
